# The Effect of Mesoporous Structure of the Support on the Oxidation of Dibenzothiophene

**DOI:** 10.3390/ijms242316957

**Published:** 2023-11-29

**Authors:** Ardian Nurwita, Maciej Trejda

**Affiliations:** Faculty of Chemistry, Adam Mickiewicz University in Poznan, Uniwersytetu Poznanskiego 8, 61-614 Poznan, Poland; ardnur@amu.edu.pl

**Keywords:** oxidative desulfurization, dibenzothiophene, SBA-15, MCF, organosilane species

## Abstract

A source of Brønsted acid centers, generated on the surface of two mesoporous silica supports of different structures (SBA-15 and MCF), was 3-(trihydroxysilyl)-1-propanesufonic acid (TPS). The materials obtained were characterized and applied as catalysts for the oxidative desulfurization of dibenzothiophene (DBT) with hydrogen peroxide as a model ODS (oxidative desulfurization) process. The properties of the materials were examined via nitrogen physisorption, XRD (X-ray Diffraction) and elemental analysis showing the preservation of the support structure after modification with organosilane species. Due to the aggregation of catalyst particles in the reaction mixture, the SBA-15 based catalyst was not very effective in DBT oxidation. Contrary, TPS/MCF catalyst exhibited a very good activity (almost total conversion of DBT after 1 h in optimized reaction conditions) and stability in dibenzothiophene oxidation in mild reaction conditions.

## 1. Introduction

Sulfur is a natural component of crude oil, and, consequently, it is found in its derivatives such as gasoline, diesel fuel as well as heavy oil. Fuel combustion releases sulfur dioxide, which is very harmful to humans and contributes to atmospheric pollution. Additionally, sulfur compounds lead to several issues, such as corrosion in storage tanks, pipelines, and equipment and the deactivation of catalysts in crude oil processing [1]. Many countries have implemented stringent regulations on the sulfur content in fuels. In the European Union, for instance, the maximum sulfur content in road vehicle diesel fuel significantly decreased, dropping from 3000 ppm to 350 ppm in 2000, then to 50 ppm in 2005, and further down to 10 ppm in 2010 [2]. To eliminate health and environmental hazards related to its presence, it is important to remove sulfur from crude oil derivatives through desulfurization processes. Hydrodesulfurization (HDS) is the most common method of removing sulfur from the fuel feedstock. It is based on the reaction of sulfur-containing organic compounds with hydrogen, resulting in sulfur-free compounds and H_2_S. HDS is realized using hydrogen at a high temperature ranging from 300 °C to 450 °C and a high pressure of 20 to 100 atm. In the process, the unsaturated carbon–carbon bonds may undergo hydrogenation, which deteriorates the properties of the fuel. Oxidative desulfurization (ODS) is one of the promising processes for desulfurization of fuels. Compared to conventional catalytic hydrodesulfurization, ODS can be carried out under mild reaction conditions and requires less energy [3,4]. For this purpose, the catalysts with active centers like molybdenum [5,6,7], vanadium [8,9,10], tungsten [11,12] and titanium [13,14], able to form peroxocomplexes with hydrogen peroxide, have been successfully tested. The success of the ODS process is determined by the catalyst efficiency in the oxidation, which strongly depends on the nature of the active centers and the type of support material.

Mesoporous silicas of different types are frequently chosen as a support for active centers due to relatively large surface area, uniform pore size, high pore volume, and high adsorption capacity [15]. The ease of modification of silica surface in the synthesis of mesoporous material and via post-synthesis modification procedures also enhance numerous applications of silica materials obtained for catalytic purposes [16]. One such modification procedure is the immobilization of different organosilane species that have been extensively investigated to generate Brønsted acidity on the silica surface. For this purpose, (3-mercaptopropyl)trimethoxysilane (MPTMS) has been applied as a modifier of SBA-15 (Santa Barbara Amorphous No. 15, CA, USA) [17], MCF (Mesostructured Cellular Foam) [18] or MCM-41 (Mobil Composition of Matter No. 41) [19]. In the last stage of catalyst preparation, SH species from MPTMS have to be oxidized into sulphonic ones most often using hydrogen peroxide. The obtained materials have been used as acidic catalysts, e.g., for acetalization of glycerol [20], esterification of oleic acid [21], aldol dimerization of levulinic acid [22], interesterification of glyceryl trioctanoate (GTO) [23] or were applied for further modification of mesoporous solids with Au [24].

Recently, MCM-41 type silica modified with MPTMS has been reported as a highly efficient Brønsted acid catalyst in oxidative desulfurization using hydrogen peroxide as the oxidant [25]. MCM-41 material has small mesopores and is relatively hydrothermally unstable [26]. The generation of Brønsted acid sites (BAS) using MPTMS has some important drawbacks, as it requires an additional step of thiol species oxidation after immobilization of organosilane modifier, which can also lead to its partial removal from the support. Recently, we have proposed to apply 3-(trihydroxysilyl)-1-propanesulfonic acid (TPS) as a source of BAS generated on mesoporous silicas [27,28]. These materials appeared not only active in esterification processes, but also showed a good catalytic stability, which allowed their reuse for several times. In view of the above-mentioned results, the aim of this study was to check a possible application of TPS containing mesoporous silicas in dibenzothiophene oxidation processes and to examine the role of the support porous structure on the catalytic behavior.

## 2. Results and Discussion

In this study, two types of mesoporous silicas of different structures, i.e., SBA-15 and MCF, were synthesized and functionalized with 3-(trihydroxysilyl)-1-propanesulfonic acid (TPS) using a post-synthetic method. The application of TPS species as a source of Brønsted acid sites allowed for omitting the oxidation step of thiol species, which usually has to be applied after (3-mercaptopropyl)trimethoxysilane (MPTMS) incorporation. 

### 2.1. Characterization of the Catalysts

**Figure 1:**
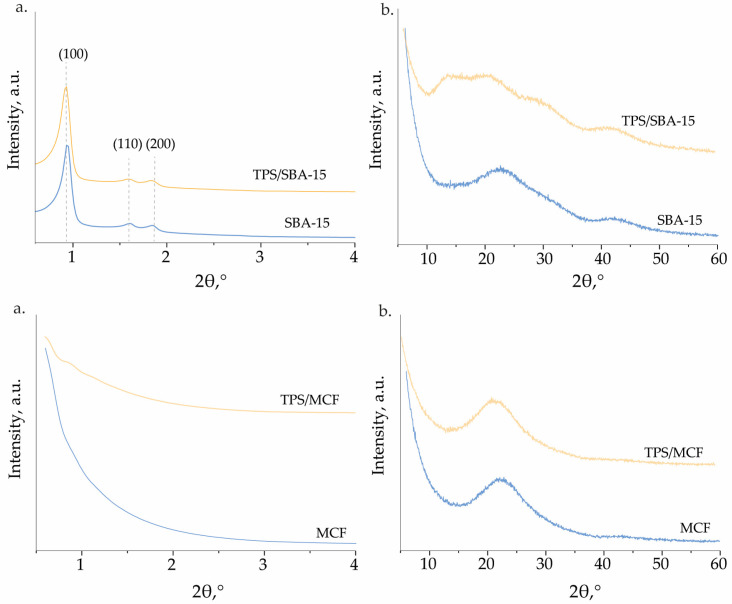
(**a**) Small-angle and (**b**) high-angle XRD pattern of the materials obtained.

The structure of SBA-15 type material was determined with XRD measurements. Figure 1 shows a small angle XRD pattern of the SBA-15 support. An intense peak at 2 theta ca. 0.9° (100) and two less intense peaks at 2 theta ca. 1.55° (110) and ca. 1.76° (200) can be observed. The presence of the first one is related to the reflections from the hexagonal plane group p6mm, characteristic of the ordered SBA-15 structure. No significant changes were observed after the incorporation of TPS species on the SBA-15 surface. This indicates that the porous hexagonal structure of the SBA-15 support is preserved after the modification procedure. The amorphous character of SBA-15 material walls was confirmed with the wide angle XRD patterns, in which no reflections coming from crystal phases are observed (Figure 1b). Figure 1 also presents the XRD patterns of MCF-based materials. Due to a much larger pore size of MCF than SBA-15 no reflections are observed in the low angle pattern, as they are shifted to smaller values of 2 theta. This is also important information indicating that the MCF support is not contaminated by the presence of SBA-15 phase, as it was presented in [29].

**Figure 2:**
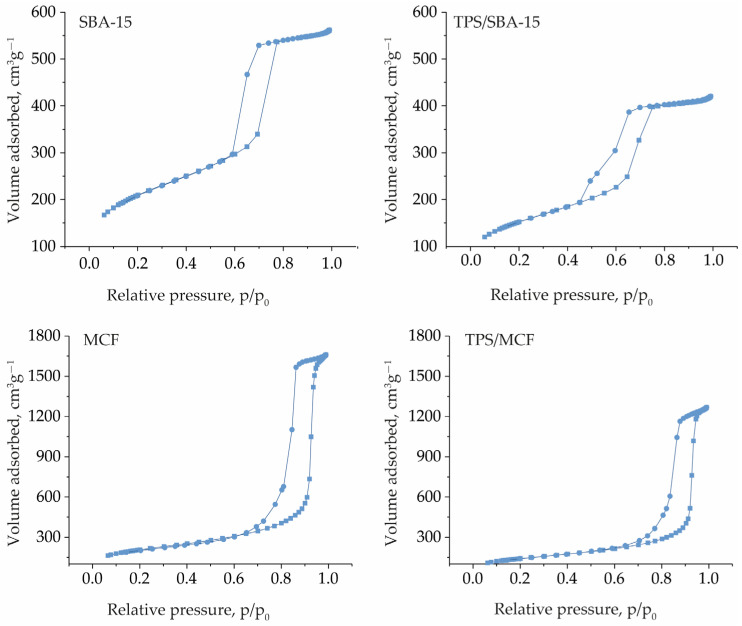
N_2_ adsorption/desorption isotherms of the materials obtained.

To characterize the textural parameters of the materials obtained, low temperature N_2_ adsorption/desorption measurements were performed. The recorded isotherms are presented in Figure 2. SBA-15 material shows a typical isotherm for ordered mesoporous silicas with relatively large pores, which is characterized by the presence of a saturation plateau and a hysteresis loop. The latter feature is observed due to the condensation of N_2_ in the pores of the material. According to IUPAC classification, this isotherm can be assigned to type IVa with H1 hysteresis loop [30]. The H1 type of hysteresis loop confirms the presence of narrow and uniform mesopores as indicated via XRD measurements. The same type of isotherm was observed after the modification of SBA-15 support with TPS; however, the volume of the adsorbed N_2_ decreased. Moreover, a discrete change in the hysteresis loop was observed. For TPS/SBA-15 material, the desorption part of hysteresis loop shifts to a lower value of p/p_0_. Similar feature has been previously observed for SBA-15 modified with TPS, and the hysteresis loop was assigned as type H5 [27]. This distinctive type of hysteresis loop is assigned to certain pore structures containing both open and partially blocked mesopores [30]. Thus, one should expect some pore blockage after modification of SBA-15 with TPS species. MCF support also shows an isotherm of type IVa with a H1 hysteresis loop. However, the hysteresis loop appears at higher values of p/p_0_ than for SBA-15 sample (0.89–0.95 vs. 0.65–0.75). This feature is characteristic of MCF materials and implies larger pore size in MCF than in SBA-15. The incorporation of TPS into MCF support leads also to a decrease in the adsorbed N_2_ volume; however, the shape of hysteresis loop does not change as observed for the TPS/SBA-15 material.

The textural parameters of the materials obtained were determined based on low temperature N_2_ adsorption/desorption measurements and are presented in Table 1. The pore size distributions in SBA-15 and MCF-based materials are presented in Figure S1 and Figure S2, respectively. Both mesoporous supports show comparable and relatively large surface areas, i.e., 762 m^2^g^−1^ and 753 m^2^g^−1^ for SBA-15 and MCF, respectively. However, they differ in pore volume, which is much bigger for MCF samples. This is in line with the structure of MCF, i.e., the presence of large interconnected cells. As described above, the incorporation of TPS species on the surface of the supports has no negative impact on their mesoporous structure. However, a decrease in the surface area and pore volume was observed after the immobilization of TPS species. Moreover, for SBA-15 support, the incorporation of TPS species led to a distinct decrease in pore size (from 10.1 nm to 8.3 nm), which was not observed after the modification of the MCF sample. For the latter material, only a slight decrease in the size of the interconnecting windows was observed.

**Figure 3:**
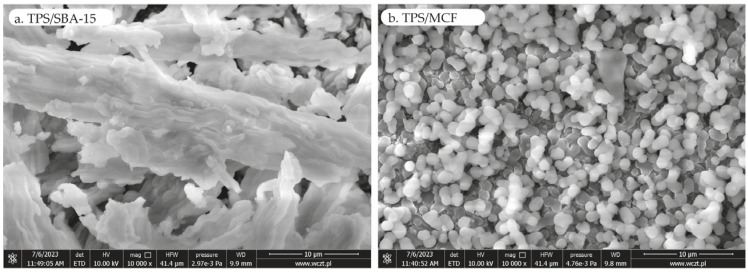
SEM images of (**a**) TPS/SBA-15 and (**b**) TPS/MCF.

The SEM images of the material obtained are presented in Figure 3. In the SEM images of TPS/SBA-15, the characteristic morphology of SBA-15 is that it forms extended worm-like fibrous bundles. In contrast, the MCF particles exhibit a spherical morphology, as observed in the micrographs.

Successful incorporation of TPS into the SBA-15 and MCF supports was confirmed with elemental analysis. The sulfur content in the materials after modification with TPS is shown in Table 1. The sulfur loading for TPS/SBA-15 and TPS/MCF was 2.2 wt.% and 2.4 wt.%, which corresponds to 0.70 mmol g^−1^ and 0.75 mmol g^−1^ of TPS, respectively.

As mentioned before, the asset of TPS application as a source of Brønsted acid sites is the presence of SO_3_H groups in the TPS molecules. Finally, the oxidation of SH groups is not necessary as in the case of MPTMS application. Moreover, it should be taken into account that the use of the latter modifier does not guarantee that all SH groups would be oxidized to SO_3_H ones. Nevertheless, for TPS modifier, the amount of sulfur in the sample after modification corresponded to the amount of sulfonic species, and thus to the number of Brønsted acid sites. The presence of Brønsted acid sites was also confirmed with pyridine adsorption followed using FTIR measurements. The spectra after pyridine adsorption on the surface of both TPS-containing materials as well as after outgassing at 150 °C and 200 °C are presented in Figure S3. The band at 1545 cm^−1^ indicated the formation of pyridinium ion on Brønsted acid sites. As one can expect for silica supports, the band corresponding to the interaction of pyridine with Lewis acid sites at 1450 cm^−1^ was not observed [31]. Pyridinium ions were also observed after outgassing of samples at 150 °C and further increase in outgassing temperature up to 200 °C caused the desorption of pyridine.

### 2.2. Oxidative Desulfurization of Dibenzothiophene

**Figure 4:**
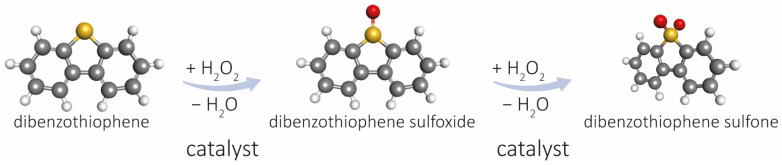
Reaction schema of catalytic oxidative desulfurization of DBT.

The catalytic activity of the materials obtained in oxidative desulfurization in liquid phase was tested for a model reaction using a mixture of dibenzothiophene (DBT, 500 ppm) in dodecane and hydrogen peroxide as an oxidizing agent. As shown in Figure 4, the transformation of benzothiophene leads to the formation of benzothiophene sulfone via benzothiophene sulfoxide.

**Figure 5:**
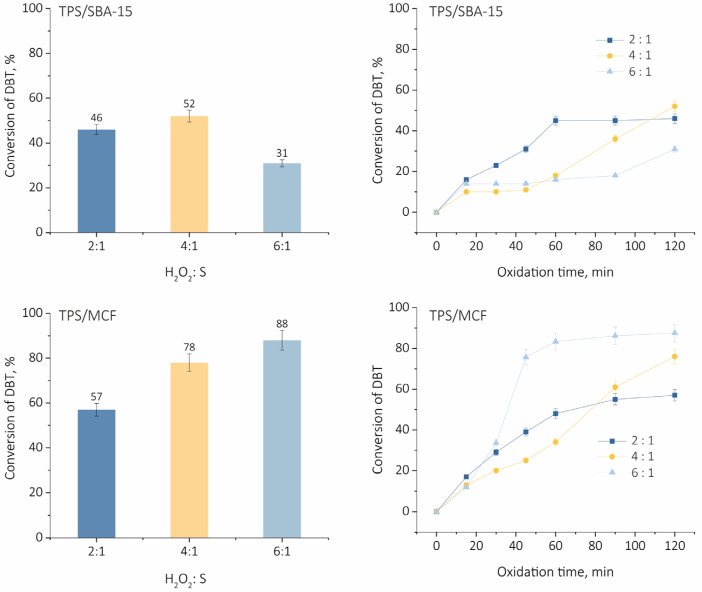
Influence of hydrogen peroxide amount on DBT conversion. Oxidation condition: w(cat.) = 1.0%, 60 °C, 1000 rpm, 120 min.

At first, the impact of hydrogen peroxide amount on the catalytic activity in oxidative desulfurization of DBT was evaluated at 60 °C for both SBA-15 and MCF-based catalysts. The reaction performed without the addition of catalyst allowed for a maximum 4% of DBT conversion after 2 h. The impact of the hydrogen peroxide concentration on the conversion of DBT is shown in Figure 5.

According to the reaction stoichiometry, two moles of hydrogen peroxide are required for a complete transformation of DBT to dibenzothiophene sulfone and only this product was observed after the reaction. For the TPS/SBA-15 catalyst, the best efficiency in oxidative desulfurization was observed for the lowest ratio of H_2_O_2_/DBT equal to 2. Only after 2 h of the reaction a slightly greater conversion of DBT was noted for the ratio of 4:1. In general, it can be concluded that the increase in hydrogen peroxide concentration has a negative impact on DBT transformation for TPS/SBA-15. It was observed that after the addition of hydrogen peroxide, the aggregation of the catalyst occurred in the reactor (Figure S4). It could be related to the addition of water to the reaction mixture together with the oxidant. Previously, it has been reported that an excess of hydrogen peroxide could inhibit ODS reaction due to the presence of water in the reactor. Water is produced during the process as a by-product of the oxidation of DBT and decomposition of hydrogen peroxide [32]. It has been also reported that the strong hydrophilicity of the catalyst leads to the aggregation of the catalyst during the fuel oxidative desulfurization process [33].

Different results were obtained when the TPS/MCF catalyst was applied. The increase in H_2_O_2_/DBT ratio from 2:1 to 4:1 led to a significant rise in DBT conversion and further increase in this ratio to 6:1 resulted in the maximum DBT conversion of 88% after 2 h of reaction. It should be mentioned that when TPS/MCF material was applied, no aggregation of catalyst particles was observed (Figure S4). This points to the less hydrophilic character of the material. Moreover, it should be taken into account that a larger pore size compared to SBA-15 support provides better diffusion of substrates and products.

Having established a much lower catalytic activity of TPS/SBA-15 and problems with the catalyst aggregation, the TPS/MCF catalyst was chosen for further study. Moreover, due to a small difference in DBT conversion for the processes carried out at the H_2_O_2_/DBT ratio of 4:1 and 6:1, a smaller concentration of hydrogen peroxide was applied.

**Figure 6:**
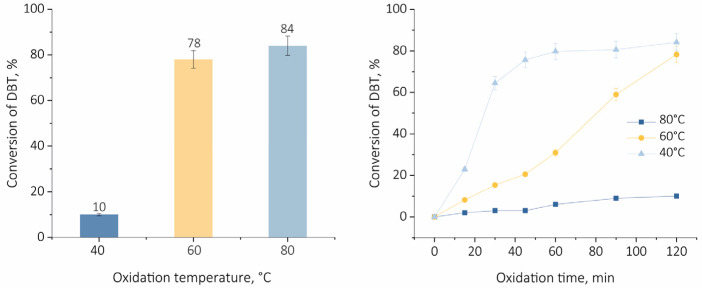
Influence of temperature on DBT conversion with TPS/MCF. Oxidation condition: H_2_O_2_/S = 4:1, w(cat.) = 1.0%, 1000 rpm, 120 min.

The impact of the temperature on the catalytic activity of TPS/MCF was evaluated. The ODS reaction was performed at different temperatures from the range 60 °C to 80 °C. The results obtained are presented in Figure 6. A relatively low DBT conversion, i.e., 10%, was measured for the reaction performed at 40 °C, whereas the increase in temperature to 80 °C allowed for obtaining an 84% of DBT conversion. It should be noticed that the increase in temperature not only causes an increase in the rate of the reaction but also influences the rate of product desorption from the surface of the catalyst [17].

**Figure 7:**
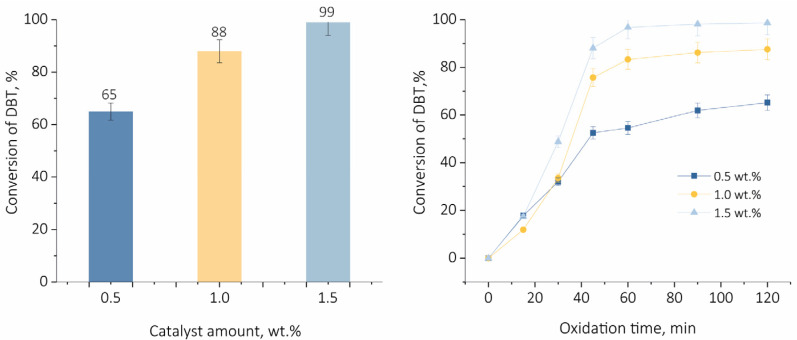
Influence of catalyst amount on DBT conversion. Oxidation condition: H_2_O_2_/S = 6:1, 80 °C, 1000 rpm, 120 min.

To study the influence of the catalyst loading on DBT conversion, the amount of the catalyst was varied in the range of 0.5 wt.% to 1.5 wt.%, relative to the mass of the model oil (Figure 7). As followed from the results, the increase in the catalyst amount entails the increase in DBT conversion. For the highest catalyst loading applied in this study, a 99% of DBT conversion was reached, which is in line with the increased number of acid sites accessible for reactants.

**Figure 8:**
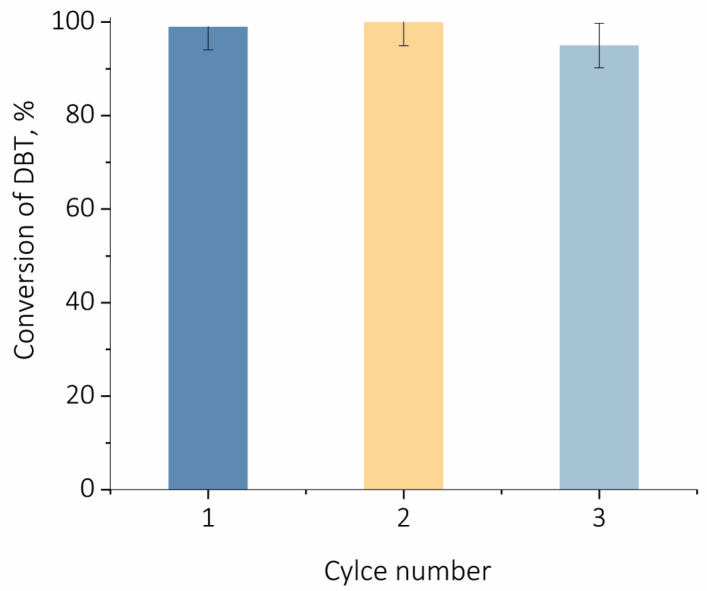
Regeneration of catalyst TPS/MCF. Oxidation condition: w(cat.) = 1.5%, H_2_O_2_/S = 6:1, 80 °C, 1000 rpm, 90 min.

To study the possibility of catalyst regeneration after the reaction, the catalyst was separated from the mixture via centrifugation, washed with acetonitrile, dried at 100 °C and reused in a new ODS cycle under the same experimental conditions. Three reaction cycles were performed, in which TPS/MCF catalyst retained its activity (Figure 8).

## 3. Materials and Methods

### 3.1. Materials and Chemicals

Tetraethyl orthosilicate (TEOS) (>99%), Pluronic P123, NH_4_F, 1,3,5-trimethylbenzene, benzothiophene, dodecane and hydrogen peroxide (30%) were purchased from Sigma-Aldrich (St. Louis, MO, USA). HCl (35%) was purchased from Stanlab (Lublin, Poland). 3-(trihydroxysilyl)-1-propanesufonic acid (30–35% in water) was purchased from Gelest (Morrisville, PA, USA).

### 3.2. Preparation of Catalysts

#### 3.2.1. Preparation of SBA-15 Support

The SBA-15 support was obtained via hydrothermal synthesis [27]. At first, the Pluronic P123 (Poly(ethylene glycol)-block-poly(propylene glycol)-block poly(ethylene glycol) (4 g) was dissolved in 150 mL of a 0.7 M solution of HCl at 40 °C. To this mixture, TEOS (8.527 g) was added dropwise with continuous stirring. Finally, the mixture was stirred at 40 °C for 20 h and then kept at 100 °C under static conditions for the next 24 h. Finally, the product was filtered off, washed with distilled water, and dried at room temperature. The template was removed via calcination at 500 °C for 6 h with a temperature ramp of 5 °C min^−1^.

#### 3.2.2. Preparation of MCF Support

MCF synthesis was performed according to the literature [34]. First, Pluronic P123 (8 g) was dissolved in 300 mL of a 0.7 M solution of HCl at 40 °C. Then, NH_4_F (0.093 g) and 1,3,5-trimethylbenzene (8 g) were added to the mixture with vigorous stirring. After 1 h, TEOS (17.054 g) was added, and the synthesis mixture was stirred for the next 20 h at 40 °C and then kept at 100 °C for 24 h in the static conditions. Finally, the solid product obtained was filtered off, washed with distilled water, and dried at room temperature. The template was removed via calcination at 500 °C for 8 h with a temperature ramp of 1 °C min^−1^.

#### 3.2.3. Preparation TPS Containing Catalysts

The modification SBA-15 and MCF with 3-(trihydroxysilyl)-1-propanesulfonic acid (TPS) was carried out according to the following procedure. A portion of 1.0 g of the support was dispersed in 30 mL of anhydrous toluene, followed by the addition of TPSA (Si/TPSA molar ratio = 10). After heating at 100 °C for 1 h, the solid was separated via filtration and washed with 75 mL of toluene, 75 mL of ethanol, and 350 mL of water. Finally, the product was dried at room temperature for 24 h. The catalyst obtained was marked as TPS/SBA-15 or TPS/MCF.

### 3.3. Catalyst Characterization

The materials were characterized via XRD measurements using a Bruker AXS D8 Advance diffractometer (Billerica, MA, USA) with Cu Kα radiation (λ = 0.154 nm). Data were collected in the low angle range of 2θ = 0.6° to 5° and in the wide angle range of 2θ = 10° to 60° with a resolution of 0.02°.

The textural properties of the catalysts were determined with N_2_ adsorption/desorption measurements using a Micromeritics ASAP 2020 instrument (Norcross, GA, USA). The specific surface areas were determined using the Brunauer–Emmett–Teller (BET) method. For the SBA-15 material, the average pore diameter was determined using the Density Functional Theory (DFT) method. The cell and window diameters of MCF material were determined from the adsorption and desorption isotherms, respectively, using the Broekhoff–de Boer–Frenkel–Fass (BdB-Fass) method.

The morphology of samples obtained was determined using SEM analysis on a Quanta 250 FEG high-resolution environmental scanning electron microscope.

The content of sulfonic groups on the support surface was determined via elemental analyses using an Elementar Analyser Vario EL III (Elemental Analysensysteme GmbH, Hanau, Germany).

Pyridine adsorption followed with FTIR measurements were performed using a Bruker INVENIO S spectrometer (Billerica, MA, USA) with an in situ vacuum cell. Prior measurement, catalysts were formed into thin wafers and placed inside the cell. The cell with the sample was then outgassed at 150 °C for 2 h. After this step, pyridine was admitted at 150 °C. After saturation with pyridine, the solid was degassed at 150 °C and 200 °C in vacuum for 30 min at each temperature. The spectrum without adsorbed pyridine (after sample outgassing at 150 °C for 2 h) was subtracted from all recorded spectra.

### 3.4. Catalytic Test

The oxidation of dibenzothiophene (DBT) was performed in a glass reactor using an EasyMax Workstation. The ODS studies were carried out using the following mixture: 500 ppm of DBT in dodecane (5 mL), and 1.0 wt.% of a catalyst and H_2_O_2_ (30%; H_2_O_2_/S = 6). The standard process was run for 120 min at 60 °C. To study the process in detail, different factors were analyzed: reaction time, amount of hydrogen peroxide, temperature, and amount of the catalyst. The samples for analysis were taken from the reaction mixture using a micro syringe. The conversion of DBT was determined using the following equation:

(1) $$\%\;\mathrm{Conversion}\;\mathrm{of}\;\mathrm{DBT}=\frac{{(\;C}_{0}-\;C\;)}{C_{0}}\times \;100\%$$

A GC (Thermo Scientific, Waltham, MA, USA) equipped with a 30 m DB-1 column and an FID detector was used to determine DBT concentration during the ODS process. Helium was applied as a carrier gas. The temperatures of the injector, and the detector were set to 250 °C and 280 °C, respectively. The initial temperature of the column was set to 80 °C for 3 min and then increased to 300 °C (temperature ramp of 10 °C min^−1^).

## 4. Conclusions

In this work, the 3-(trihydroxysilyl)-1-propanesulfonic acid (TPS)-containing catalysts based on SBA-15 and MCF were synthesized. Successful incorporation of TPS into the SBA-15 and MCF supports was confirmed with elemental analysis; the results were in agreement with those obtained using the pyridine adsorption method combined with the FTIR adsorption measurements. The results indicated that TPS/SBA-15 and TPS/MCF catalysts contain Brønsted acid sites. The use of the catalyst TPS/SBA-15 was observed to be accompanied by aggregation of the particle catalyst in the reactor, thereby significantly limiting the activity in DBT oxidation. On the other hand, the TPS/MCF catalyst was found to be an effective catalyst for these processes. Up to 99% DBT conversion was achieved under the following conditions: w(cat.) = 1.5%, H_2_O_2_/S = 6:1, 80 °C, 1000 rpm, and 1.5 h; this catalytic activity was maintained over consecutive cycles. Moreover, it was shown that the structure and nature of the catalyst’ support determine the catalytic activity in DBT oxidation. This is an important feature which should be taken into account in designing the catalyst dedicated to ODS processes.

## Figures and Tables

**Table 1:** Textural properties of the materials obtained.

Catalyst	S_BET_ (m^2^ g^−1^) ^a^	Pore Size (nm)	V_Total_ (cm^3^ g^−1^) ^e^	S Content, (wt.%)
SBA-15	762	10.1 ^b^	0.83	-
TPS/SBA-15	554	8.3 ^b^	0.64	2.2 ^f^
MCF	753	23.7 ^c^; 12.0 ^d^	2.21	-
TPS/MCF	517	24.0 ^c^; 11.6 ^d^	1.72	2.4 ^f^

^a^ S_BET_—the specific surface area determined by the BET method. ^b^ Pore size—pore size distribution (PSD) calculated by the DFT method. ^c^ Cell diameter—PSD from adsorption of N_2_ isotherm (BdB-Fass method). ^d^ Window diameter—PSD from desorption of the N_2_ isotherm (BdB-Fass method). ^e^ V_Total_—total pore volume—BJH adsorption cumulative volume of pores. ^f^ S content—sulfur content by elemental analysis.

## Data Availability

The data presented in this study are available on request from the corresponding author via e-mail.

## Supplementary Materials

The following supporting information can be downloaded at: https://www.mdpi.com/article/10.3390/ijms242316957/s1.

## References

[1] Ahmadian M., Anbia M. (2021). Oxidative Desulfurization of Liquid Fuels Using Polyoxometalate-Based Catalysts: A Review. Energy Fuels.

[2] Rajendran A., Cui T.Y., Fan H.X., Yang Z.F., Feng J., Li W.Y. (2020). A comprehensive review on oxidative desulfurization catalysts targeting clean energy and environment. J. Mater. Chem. A.

[3] Boshagh F., Rahmani M., Rostami K., Yousefifar M. (2022). Key Factors Affecting the Development of Oxidative Desulfurization of Liquid Fuels: A Critical Review. Energy Fuels.

[4] Haghighi M., Gooneh-Farahani S. (2020). Insights to the oxidative desulfurization process of fossil fuels over organic and inorganic heterogeneous catalysts: Advantages and issues. Environ. Sci. Pollut. Res..

[5] Chen Y., Tian Q., Tian Y., Cui J., Wang G. (2021). Ultra-Deep Oxidative Desulfurization of Fuel with H_2_O_2_ Catalyzed by Mesoporous Silica-Supported Molybdenum Oxide Modified by Ce. Appl. Sci..

[6] Teimouri A., Mahmoudsalehi M., Salavati H. (2018). Catalytic oxidative desulfurization of dibenzothiophene utilizing molybdenum and vanadium oxides supported on MCM-41. Int. J. Hydrog. Energy.

[7] Akopyan A., Polikarpova P., Gul O., Anisimov A., Karakhanov E. (2020). Catalysts Based on Acidic SBA-15 for Deep Oxidative Desulfurization of Model Fuels. Energy Fuels.

[8] Rivoira L.P., Cussa J., Martínez M.L., Beltramone A.R. (2020). Experimental design optimization of the ODS of DBT using vanadium oxide supported on mesoporous Ga-SBA-15. Catal. Today.

[9] Wang Y., Zhang G., Guan T., Xu F., Wu J., Zhou E., Wang J., Li K. (2020). Ultra-Deep Oxidative Desulfurization of Model Oil Catalyzed by In Situ Carbon-Supported Vanadium Oxides Using Cumene Hydroperoxide as Oxidant. Chem. Sel..

[10] Ramos J.M., Wang J.A., Flores S.O., Chen L., Arellano U., Noreña L.E., González J., Navarrete J. (2021). Ultrasound-Assisted Hydrothermal Synthesis of V_2_O_5_/Zr-SBA-15 Catalysts for Production of Ultralow Sulfur Fuel. Catalysts.

[11] Polikarpova P., Akopyan A., Shigapova A., Glotov A., Anisimov A., Karakhanov E. (2018). Oxidative Desulfurization of Fuels Using Heterogeneous Catalysts Based on MCM-41. Energy Fuels.

[12] Estephane G., Lancelot C., Blanchard P., Dufaud V., Chambrey S., Nuns N., Toufaily J., Hamiye T., Lamonier C. (2019). W-SBA based materials as efficient catalysts for the ODS of model and real feeds: Improvement of their lifetime through active phase encapsulation. Appl. Catal. A.

[13] Wang Y., Du F., Wang C., Zhao J., Sun H., Sun C. (2022). The synthesis and oxidation desulfurization performance of Ti-modified hierarchical cheese-like ZSM-5 zeolite. J. Chem. Res..

[14] Rivoira L.P., Ledesma B.C., Juárez J.M., Beltramone A.R. (2018). Novel and simple one-pot method for the synthesis of TiO_2_ modified-CMK-3 applied in oxidative desulfurization of refractory organosulfur compounds. Fuel.

[15] Shinde P.S., Suryawanshi P.S., Patil K.K., Belekar V.M., Sankpal S.A., Delekar S.D., Jadhav S.A. (2021). A brief overview of recent progress in porous silica as catalyst supports. J. Compos. Sci..

[16] Liang Y. (2021). Recent advanced development of metal-loaded mesoporous organosilicas as catalytic nanoreactors. Nanoscale Adv..

[17] Zhao D., Huo Q., Feng J., Chmelka B.F., Stucky G.D. (1998). Nonionic Triblock and Star Diblock Copolymer and Oligomeric Surfactant Syntheses of Highly Ordered, Hydrothermally Stable, Mesoporous Silica Structures. J. Am. Chem. Soc..

[18] Lettow J.S., Han Y.J., Schmidt-Winkel P., Yang P., Zhao D., Stucky G.D., Ying J.Y. (2000). Hexagonal to Mesocellular Foam Phase Transition in Polymer-Templated Mesoporous Silicas. Langmuir.

[19] Beck J.S., Vartuli J.C., Roth W.J., Leonowicz M.E., Kresge C.T., Schmitt K.D., Chu C.T.W., Olson D.H., Sheppard E.W., McCullen S.B. (1992). A New Family of Mesoporous Molecular Sieves Prepared with Liquid Crystal Templates. J. Am. Chem. Soc..

[20] Castanheiro J.E., Vital J., Fonseca I.M., Ramos A.M. (2022). Acetalization of glycerol with hexanal in the presence of SBA-15 with sulfonic acid groups. Catal. Today.

[21] Cabrera-Munguia D., González H., Tututi-Ríos E., Gutiérrez-Alejandre A., Rico J. (2018). Acid properties of M-SBA-15 and M-SBA-15-SO3H (M = Al, Ti) materials and their role on esterification of oleic acid. J. Mater. Res..

[22] Paniagua M., Cuevas F., Morales G., Melero J.A. (2021). Sulfonic Mesostructured SBA-15 Silicas for the Solvent-Free Production of Bio-Jet Fuel Precursors via Aldol Dimerization of Levulinic Acid. ACS Sustain. Chem. Eng..

[23] Testa M.L., Tummino M.L., Venezia A.M., Russo M. (2023). Interesterification of Glyceryl Trioctanoate Catalyzed by Sulfonic Silica-Based Materials: Insight into the Role of Catalysts on the Reaction Mechanism. Materials.

[24] Wisniewska J., Dziedzic I., Ziolek M. (2020). A platinum promoted Ag/SBA-15 catalyst effective in selective oxidation of methanol design and surface characterization. RSC Adv..

[25] Polikarpova P., Akopyan A., Shlenova A., Anisimov A. (2020). New mesoporous catalysts with Brønsted acid sites for deep oxidative desulfurization of model fuels. Catal. Commun..

[26] Habeche F., Hachemaoui M., Mokhtar A., Chikh K., Benali F., Mekki A., Zaoui F., Cherifi Z., Boukoussa B. (2020). Recent Advances on the Preparation and Catalytic Applications of Metal Complexes Supported-Mesoporous Silica MCM-41 (Review). J. Inorg. Organomet. Polym. Mater..

[27] Trejda M., Drobnik M., Nurwita A. (2021). Application of microwave radiation in the grafting of acidic sites on SBA-15 type material. J. Porous Mater..

[28] Trejda M., Kaszuba A., Nurwita A., Ziolek M. (2021). Towards Efficient Acidic Catalysts via Optimization of SO_3_H-Organosilane Immobilization on SBA-15 under Increased Pressure: Potential Applications in Gas and Liquid Phase Reactions. Materials.

[29] Stawicka K., Sobczak I., Trejda M., Sulikowski B., Ziolek M. (2012). Organosilanes affecting the structure and formation of mesoporous cellular foams. Microporous Mesoporous Mater..

[30] Thommes M., Kaneko K., Neimark A.V., Olivier J.P., Rodriguez-Reinoso F., Rouquerol J., Sing K.S.W. (2015). Physisorption of gases, with special reference to the evaluation of surface area and pore size distribution (IUPAC Technical Report). Pure Appl. Chem..

[31] Zholobenko V., Freitas C., Jendrlin M., Bazin P., Travert A., Thibault-Starzyk F. (2020). Probing the acid sites of zeolites with pyridine: Quantitative AGIR measurements of the molar absorption coefficients. J. Catal..

[32] Caero L.C., Hernández E., Pedraza F., Murrieta F. (2005). Oxidative desulfurization of synthetic diesel using supported catalysts: Part I. Study of the operation conditions with a vanadium oxide-based catalyst. Catal. Today.

[33] Akopyan A.V., Polikarpova P.D., Arzyaeva N., Anisimov A.V., Maslova O., Senko O., Efremenko E.N. (2021). Model Fuel Oxidation in the Presence of Molybdenum-Containing Catalysts Based on SBA-15 with Hydrophobic Properties. ACS Omega.

[34] Stawicka K., Decyk P., Wojtaszek-Gurdak A., Ziolek M. (2019). Comparative study of acid-basic properties of MCF impregnated with niobium and cerium species. Catal. Today.

