# Supplementary material for: The Effect of Mesoporous Structure of the Support on the Oxidation of Dibenzothiophene
Source: Int J Mol Sci. 2023 Nov 29;24(23):16957. doi: 10.3390/ijms242316957 (PMC10707218; doi:10.3390/ijms242316957)
Supplement: Supplementary file 1 [file ijms-24-16957-s001.zip › ijms-2696633-supplementary.pdf]

Article

The Effect of Mesoporous Structure of the Support on the Oxidation of Dibenzothiophene

Ardian Nurwita and Maciej Trejda *

Faculty of Chemistry, Adam Mickiewicz University in Poznan, Uniwersytetu Poznanskiego 8, 61-614 Poznan, Poland; ardnur@amu.edu.pl
* Correspondence: tmaciej@amu.edu.pl; Tel.: +48-61-8291686

Supplementary Materials

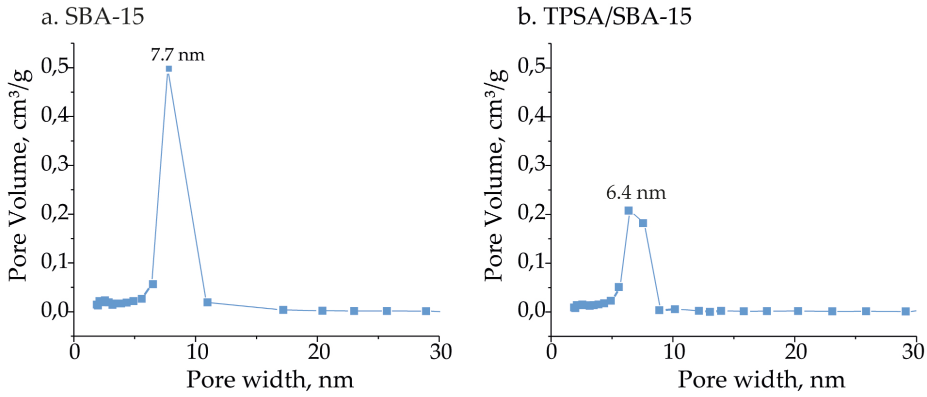

Figure S1: Pore size distribution estimated from DFT of SBA-15 and TPS/SBA-15 materials.

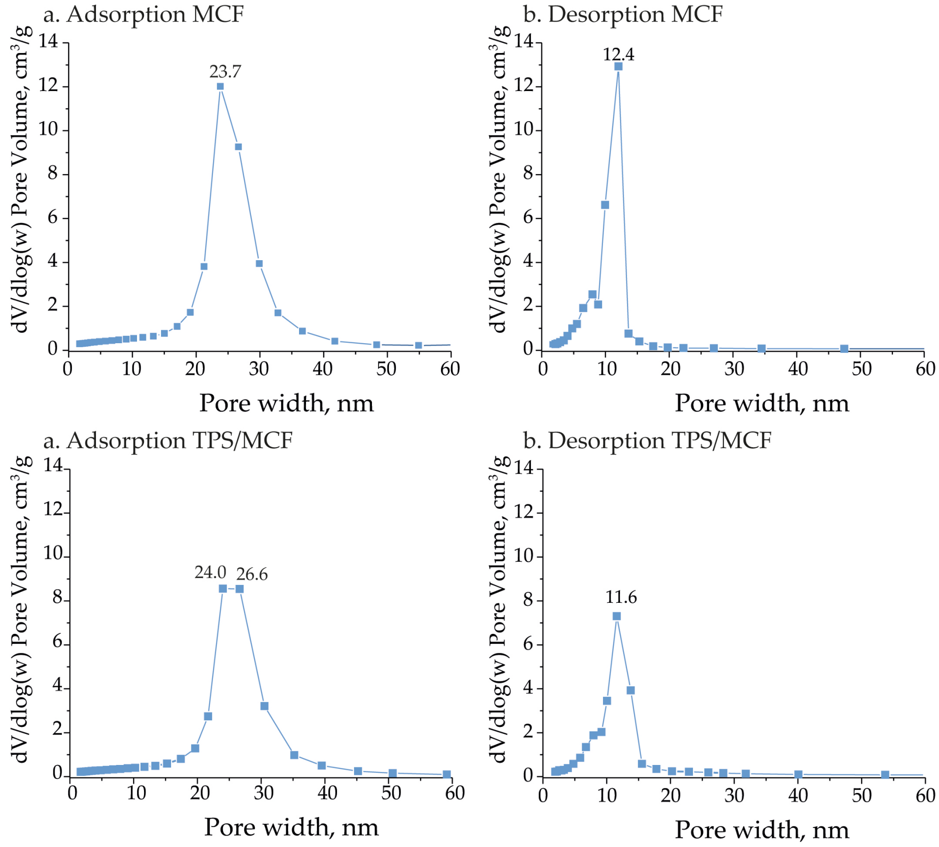

Figure S2. Pore size distribution estimated from (a) adsorption and (b) desorption branches of N_2 adsorption/desorption isotherms of MCF and TPS/MCF materials.

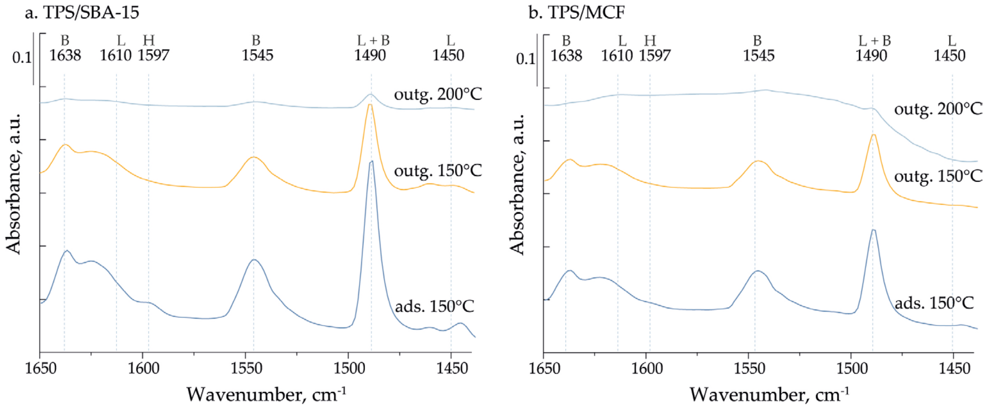

Figure S3. The spectra of TPS/SBA-15 and TPS/MCF after pyridine adsorption at 150°C and after outgassing for 30 min. at 150°C and 200°C.

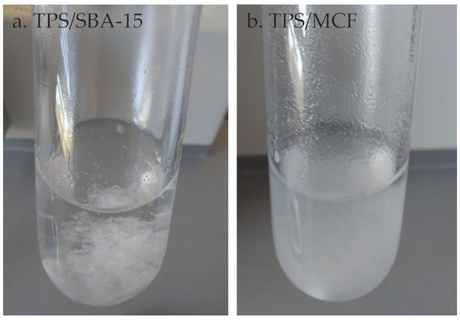

Figure S4. Image of TPS/SBA-15 (left) and TPS/MCF (right) catalyst during the ODS.
